# Significance of long non-coding RNA IFNG-AS1 in the progression and clinical prognosis in colon adenocarcinoma

**DOI:** 10.1080/21655979.2021.2003944

**Published:** 2021-12-07

**Authors:** Zhaoshun Wang, Zhongzheng Cao, Zhen Wang

**Affiliations:** a Department of Anorectal Surgery, Weifang People’s Hospital, Shandong, China; b Department of Anorectal Surgery, Zibo First Hospital, Shandong, China

**Keywords:** LncRNA IFNG-AS1, diagnosis, prognosis, colon adenoma, progression

## Abstract

Colon adenocarcinoma originates from adenoma and triggers serious healthy burdensome. lncRNAs develop a crucial role in the progression of colorectal carcinoma. In this study, we aimed to investigate the clinical value and potential role of lncRNA interferon (IFN) gamma antisense RNA 1 (IFNG-AS1) in colon adenocarcinoma. This study enrolled 95 colorectal adenoma patients, 128 colorectal adenocarcinoma patients, and 88 healthy individuals. The serum, tissue IFNG-AS1 expression levels were explored by real-time quantitative reverse transcription-PCR (RT-qPCR) assay. The receiver operator characteristic curve and Kaplan-Meier method were used to assess the clinical significance of IFNG-AS1. The chi-square test was used to analyze the association between tissue IFNG-AS1 and clinical characteristics. Functional experiments were conducted to delve into the effects of IFNG-AS1 on cellular activities (cell viability/migration/invasion). The target miRNA of IFNG-AS1 was also explored. IFNG-AS1 expression in both serum and tissue samples was elevated in patients. Serum IFNG-AS1 could diagnose colon adenoma and adenocarcinoma patients from the healthy control. High tissue IFNG-AS1 was correlated with several clinical characteristics and a shorter overall survival time. Silence of IFNG-AS1 could be available for repressing cellular capacities via the sponge to miR-627-3p. IFNG-AS1 was rised in colon adenocarcinoma and it was relevant to tumor size, TNM stage, and poor prognosis of patients. Beyond that, downregulated expression of IFNG-AS1 may repress malignant progression of colon adenocarcinoma by regulating miR-627-3p. IFNG-AS1 might be a potential diagnosis or prognosis predictor for colon adenocarcinoma patients.

## Introduction

Colorectal cancer, as one of the most common gastrointestinal malignancies, has an increasing incidence and mortality rate with each passing day [1]. It is reported that most colorectal carcinomas have undergone a normal mucous membrane-adenoma-adenocarcinoma development process [2]. Adenocarcinoma is the most crucial histologic type of colorectal carcinoma and adenoma is identified as the most important precancerous lesion of colorectal adenocarcinoma [3,4]. It is difficult to diagnose colorectal adenocarcinoma in the early stage, so most patients are diagnosed at middle or advanced stages for primary care. Although the advanced diagnosis and treatment technology, the postoperative survival rate of patients has not improved significantly [5]. The lack of effective diagnostic markers or methods to achieve early diagnosis and treatment of colorectal adenocarcinoma and its precancerous lesions is one of the main causes [6]. Early detection and removal of colorectal adenocarcinoma and its adenomatous lesions will be conducive to reducing morbidity and mortality [7]. As a result, there is great practical meaning to manifest the molecular mechanism of the occurrence of colorectal adenocarcinoma for the sake of improving the early detection and disease treatment.

RNA is transcribed from DNA, which can be divided into coding RNA (mRNAs) and non-coding RNAs (ncRNAs) [8]. Non-coding RNAs contain long ncRNAs (lncRNAs) and short microRNAs (miRNAs) [9]. lncRNAs with a transcript length of more than 200 nucleotides have no protein-coding ability, which can regulate gene expression by interacting with RNA, DNA, and protein [10]. The aberrant lncRNA expression is commonly found in multiple human tumors, such as breast cancer [11], lung cancer [12], and colon cancer [13]. lncRNA interferon (IFN) gamma antisense RNA 1 (IFNG-AS1) also called *TMEVPG1* or *NeST*, was first reported in the immune system of diseases and acted crucial oncogenic role in several types of cancers, such as pituitary adenomas [14], breast cancer [15], and acute leukemia [16]. Recently, a study reported that IFNG-AS1 was overexpressed in ulcerative colitis, which can exert an impact on the colon and large intestine [17]. Nevertheless, whether IFNG-AS1 was abnormally expressed in colon adenoma and adenocarcinoma remains unknown.

In the current paper, the aberrantly expressed IFNG-AS1 was discovered and verified in colon adenoma and adenocarcinoma patients. It was also discovered that IFNG-AS1 expression in tumor tissues was associated with tumor size and TNM stage, as well as had clinical significance. Last but not least, the functional role and mechanism of IFNG-AS1 in colon adenocarcinoma cells were also pinpointed.

## Materials and methods

### Patients and samples

In this study, 95 colorectal adenoma patients, 128 colorectal adenocarcinoma patients, and 88 healthy individuals were enrolled from February 2013 to May 2015 in Weifang People’s Hospital. The healthy controls (HC) were healthy subjects from the physical examination center of the hospital, whose physical examination report has no obvious abnormality and the age and sex were matched with the colorectal adenoma and adenocarcinoma group. The serum samples were collected from these patients and healthy individuals and stored for RNA extraction. And the 128 pairs of tumor tissues and adjacent normal tissues (distance from lesion sites > 5 cm and no tumor infiltration was confirmed by pathology) were gathered from colorectal adenocarcinoma patients during surgery and stored at liquid nitrogen until use. The cases’ inclusion criteria were: 1) The patient was diagnosed with colorectal adenocarcinoma, and the relevant medical history and treatment were complete without missing, 2) Surgical treatment was performed for the first time, and no relevant intervention measures such as radiotherapy, chemotherapy, or targeted drug therapy were implemented before surgery, 3) The preoperative examination revealed no other tissue or organ malignancy. For the clinical analysis, the relevant medical clinical characteristics information and five-year overall survival data were collected and recorded for analysis.

This experiment was approved by the Ethics Committee of Weifang People’s Hospital before implementation, and the enrolled participants have been informed of the experimental content in writing before sample collection, and the written informed consents signed by the participant have been obtained.

### Cell culture

The cells used in this study were human colonic epithelial cell lines NCM 460 and colorectal cancer cell lines HT-29, HCT-15, SW480, and SW1116. Among them, NCM 460 cell lines were provided from the Union Cell Bank, Chinese Academy of Medical Sciences, HT-29, HCT-15, SW480, and SW1116 cell lines were all purchased from the American Type Culture Collection (ATCC; Manassas, VA, USA). The cell lines were cultured in a DMEM medium (Gibco, Carlsbad, USA) with 10% FBS (Gibco) as the supplements and allowed to propagate in the 5% CO_2_ humidified incubator at 37°C.

### lncRNA siRNA and miRNAs transfection

The transfection reagents IFNG-AS1 siRNA (si-IFNG-AS1) and siRNA negative control (siRNA-NC), miR-627-3p mimic, miR-627-3p inhibitor, and corresponding NCs (mimic NC and inhibitor NC) were all obtained from GenePharma (Shanghai, China). After being incubated in 6-well plates and grown to a cell density of 50%, the cells were transfected with the transfection reagents for 24 h with the help of Lipofectamine 2000 reagent [18]. Transfection efficiency was measured using reverse transcription-quantitative PCR (RT-qPCR) 48 h post-transfection. The control group was the untreated cells.

### RNA isolation and RT-qPCR assay *[19]*

Serum, tissue samples, and cell lines were treated with TRIzol reagent (Invitrogen; Thermo Fisher Scientific) to extract total RNA per the manufacturer’s protocol. cDNA was then synthesized by the PrimeScript RT reagent kits (Takara) for lncRNA and a miScript SYBR® Green PCR kit (Qiagen, Germany) for miRNA. All PCR reactions were run on Applied Biosystem 7500 Real-Time PCR system in accordance with the manufacturer’s instructions. The internal control was GAPDH or U6 for normalization of the relative expression of each gene with the formula 2^−ΔΔCt^.

### Cell counting Kit-8 (CCK-8) assay

Cell viability in response to IFNG-AS1 silence was assessed using a CCK-8 kit (Dojindo, Japan) [20]. The cells (1000 cells/well) were incubated in 96-well plates and detected at 0, 24, 48, and 72 h time points. Then, a 10 μl CCK-8 kit was added and the cells were incubated for an additional 2 h at 37°C. The absorbance values were determined with a microplate reader (BioTek Instruments, USA) at 450 nm.

### Transwell migration and invasion assay

HT-29 (1 × 10^5^ cells/well) and SW480 cells (2 × 10^5^ cells/well) that were re-suspended in DMEM medium without FBS were plated in the top, uncoated Matrigel Transwell chambers (8 μm pore size; Corning, USA) for migration assay, while plated in the top Transwell chambers that pre-coated with Matrigel™ Matrix (BD Biosciences) for invasion assay. The culture medium with 10% FBS was added in the bottom chamber to induce cell migration or invasion. After 24 h of incubation, the migratory or invasive cells in the lower chamber were fixed and stained. The cells were counted under a light microscope in five random fields of view.

### Dual-luciferase reporter assay

DIANA tools LncBase Experimental v.2 (http://carolina.imis.athena-innovation.gr/diana_tools/web/index.php?r=lncbasev2%2findex-experimental) was used to predict potential target miRNAs and the association between IFNG-AS1 and miR-627-3p. The wild-type (WT) or mutated (MUT) fragment of IFNG-AS1 containing the target sequences of miR-627-3p was inserted into a pmirGLO reporter vector (Promega, Fitchburg, WI, USA) to form the WT-IFNG-AS1 or MUT-IFNG-AS1. Then the cells were co-transfected with WT-IFNG-AS1 or MUT-IFNG-AS1 and miR-627-3p mimic, inhibitor, or the NCs for 24 h with the help of Lipofectamine 2000. The firefly luciferase activity was detected using the Dual-Luciferase Reporter assay system (Promega Corporation).

### Statistical analysis

Each experiment was repeated at least three times and data were presented as the mean ± SD. All analyses were carried out using SPSS software (version 20.0; IBM, Armonk, NY) and Graphpad Prism software (version 7.0; GraphPad, San Diego, CA). Significant differences of groups were calculated using paired Student’s t-test or one-way/two-way ANOVA followed by Tukey’s post-hoc test. The correlation analysis was carried out by the Pearson correlation tests. The clinical diagnostic or prognostic values of IFNG-AS1 were evaluated using receiver operator characteristic (ROC) curve or Kaplan-Meier curve with log-rank test, as well as multivariate Cox regression analysis. The *P*-value small than 0.05 was considered statistically significant for analysis.

## Results

To explore the clinical significance and functional role of IFNG-AS1 in colon adenocarcinoma, the relative expression of IFNG-AS1 was measured using RT-PCR in healthy control, colon adenoma patients, and adenocarcinoma patients. ROC curve and Kaplan-Meier curves were conducted to evaluate the clinical significance of IFNG-AS1. The functional experiments including CCK-8 assay and Transwell assays were carried out to investigate the functional role of IFNG-AS1 in colon adenocarcinoma. Additionally, the dual-luciferase reporter assay confirmed the potential target of IFNG-AS1.

### IFNG-AS1 expression and detective values in colorectal adenoma and adenocarcinoma patients

**Figure 1.**
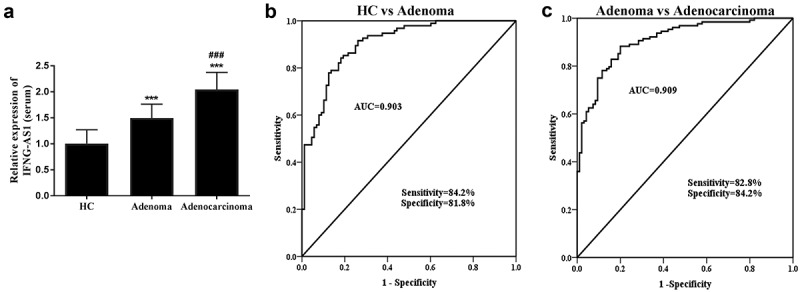
Serum IFNG-AS1 expression in three groups (healthy control, colon adenoma, and adenocarcinoma patients). (a) Serum IFNG-AS1 expression was higher in colon adenocarcinoma patients than in colon adenoma patients and healthy controls. ***P < 0.001; ^###^*P* < 0.001. (b) ROC curves for the ability of IFNG-AS1 to differentiate the colon adenoma patients from the healthy control group. (AUC = 0.903) (c) ROC curves for the ability of IFNG-AS1 to distinguish the colon adenocarcinoma patients from the colon adenoma patients with an AUC of 0.909

First and foremost, the relative serum IFNG-AS1 expression levels were measured via RT-qPCR assay in three groups. The results indicated that IFNG-AS1 expression levels were increased in the colon adenoma patients and adenocarcinoma patients compared with healthy control (both *P* < 0.001, Figure 1(a)). Moreover, Figure 1(a) also indicated that the IFNG-AS1 expression was higher in colorectal adenocarcinoma patients than that of adenoma patients (*P* < 0.001).

From its abnormal expression, the predictive value was evaluated via the receiver operator characteristic curve (ROC) curve. The further validation of the findings implied that the area under the ROC curve (AUC) of IFNG-AS1 in the diagnosis of colorectal adenoma from healthy individuals was 0.903 with a sensitivity of 84.2% and specificity of 81.8% (Figure 1(b)). What’s more, the AUC for the diagnosis of colon adenocarcinoma from adenoma patients was 0.909, sensitivity was 82.8%, and specificity was 84.2% (Figure 1(c)). The above-mentioned data revealed that serum IFNG-AS1 may have the diagnostic value in distinguishing both colon adenocarcinoma and adenoma patients from a healthy control.

### IFNG-AS1 expression was in correlation with overall survival in colorectal adenocarcinoma patients

**Figure 2.**
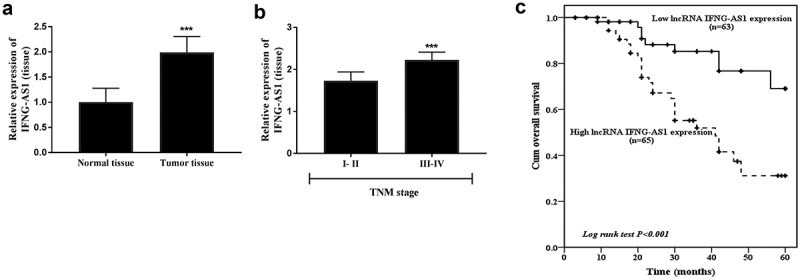
Tumor tissue IFNG-AS1 expression and its value in prognosis. (a) The IFNG-AS1 expression was increased in colon adenocarcinoma tissues compared to adjacent tissues. ***P < 0.001. (b) IFNG-AS1 expression levels were higher in III–IV stage tissues than in I–II stage tissues. ***P < 0.001. (c) Correlation between IFNG-AS1 expression and overall survival of colon adenocarcinoma patients. (*P* < 0.001)

The expression of IFNG-AS1 in tumor tissues and adjacent normal tissues was also detected. As displayed in Figure 2(a), the IFNG-AS1 expression was raised in tumor tissues (P < 0.001). According to the mean expression level, the colon adenocarcinoma patients were subdivided into low and high expression groups for further clinical analysis. The χ^2^ test analysis (Table 1) indicated that high IFNG-AS1 expression was associated with higher tumor size (*P* = 0.013) and TNM stage (*P* = 0.001). Furthermore, the IFNG-AS1 expression was analyzed in low and high TNM stage patients. It could be observed that patients with high TNM stages (III–IV) showed higher IFNG-AS1 expression levels than low TNM stages (I–II) (*P* < 0.001, Figure 2(b)).

**Table 1.** Correlation of the lnc IFNG-AS1 expression with clinical characteristics in colorectal adenocarcinoma

Parameters	lnc IFNG-AS1 expression N = 128		*P*
	Low (n = 63)	High (n = 65)	
Age			0.281
≤ 50	37	32	
> 50	26	33	
Gender			0.737
Male	34	37	
Female	29	28	
Tumor size			0.013
≤ 5 cm	38	25	
> 5 cm	25	40	
Location (colon)			0.613
Left	36	40	
Right	27	25	
Differentiation			0.110
Well, Moderate	37	29	
Poor	26	36	
TNM stage			0.001
I, II	40	23	
III, IV	23	42

The association between IFNG-AS1 and overall survival was dissected by Kaplan-Meier analysis and Cox regression analysis, and the results showed that compared with patients in the low IFNG-AS1 expression group, and patients in the high IFNG-AS1 expression group had a worse prognosis (*P* < 0.001, Figure 2(c)). The multivariate Cox analysis results indicated that IFNG-AS1 expression (*P* < 0.001) and TNM stage (*P* = 0.019) were independent prognostic risk factors (Table 2). The aforesaid data stated that IFNG-AS1 expression might be a prognostic predictor in colon adenocarcinoma.

**Table 2.** Multivariate Cox analysis of clinical characteristics in relation to overall survival

Characteristics	Multivariate analysis		
	HR	95% CI	*P*
Lnc IFNG-AS1	5.650	2.415–13.222	<0.001
Age	1.155	0.591–2.259	0.673
Gender	1.537	0.763–3.095	0.229
Tumor size	2.135	0.994–4.589	0.052
Location (colon)	0.605	0.303–1.205	0.153
Differentiation	1.785	0.900–3.542	0.097
TNM stage	2.577	1.172–5.668	0.019

### Knockdown of IFNG-AS1 weakened cell proliferation, migration, and invasion

**Figure 3.**
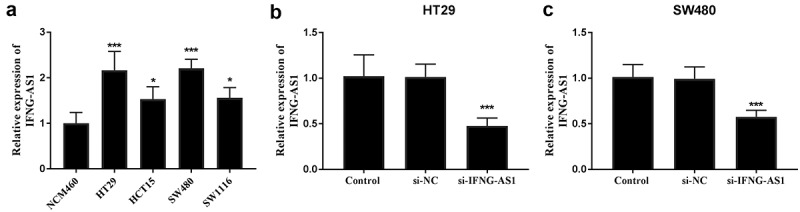
Expression of IFNG-AS1 in colon adenocarcinoma cells and cell transfection. (a) The expression of IFNG-AS1 was increased in cancer cells rather than NCM460 normal cells. *P < 0.05, ***P < 0.001. (b) si-IFNG-AS1 decreased the expression of IFNG-AS1 in HT-29 cells. ***P < 0.001. (c) The expression of IFNG-AS1 was downregulated by IFNG-AS1 siRNA. ***P < 0.001

To probe the function of IFNG-AS1 in colon adenocarcinoma, its expression levels were detected in colon adenocarcinoma cells and the IFNG-AS1 knockdown model was constructed. Among the cells, IFNG-AS1 was highly expressed in colon adenocarcinoma cells (*P* < 0.001, Figure 3(a)), especially after transfecting IFNG-AS1 siRNA in the HT-29 and SW480 cells, RT-qPCR assay was conducted to confirm the interference efficiency, and the difference showed the statistical significance (*P* < 0.001, Figure 3(b,c)).

**Figure 4.**
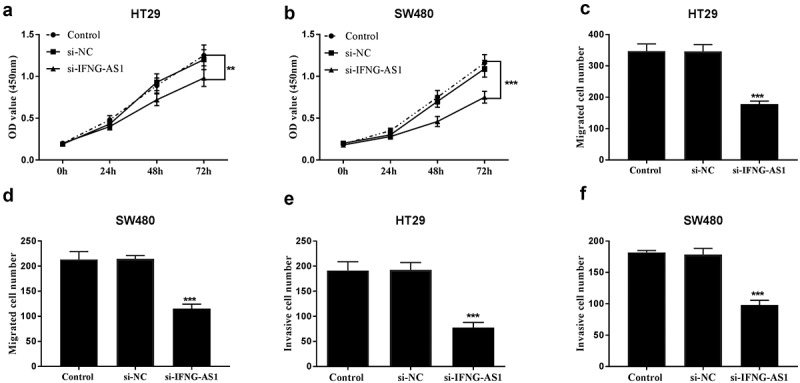
Silence of IFNG-AS1 repressed cellular behaviors. Knockdown of IFNG-AS1 decreased cell proliferation in HT-29 cells (a) and SW480 cells (b). **P < 0.01, ***P < 0.001. Decreased IFNG-AS1 inhibited cell migration in HT-29 cells (c) and SW480 cells (d). ***P < 0.001. Transwell invasion assay manifested that si-IFNG-AS1 restained cell invasion abilities in HT-29 cells (e) and SW480 cells (f). ***P < 0.001

Cell proliferative abilities were measured using CCK-8 assay in IFNG-AS1 knocking down cell lines. The results in Figure 4(a,b) demonstrated that cell proliferative capacities were decreased in IFNG-AS1 silent groups (*P* < 0.01). Cell migratory capacities were inspected by Transwell migration assay in IFNG-AS1 silent cell lines. Figure 4(c,d) implied that the migration of HT-29 and SW480 cells was reduced after being transfected with si-IFNG-AS1 (*P* < 0.001). Transwell invasion assay also manifested that the invasion abilities of HT-29 (Figure 4(e)) and SW480 cells (Figure 4(f)) with low IFNG-AS1 expression levels were suppressed (*P* < 0.001).

### Increased expression of IFNG-AS1 is relevant to the decrease of miR-627-3p expression in HT-29 cells

**Figure 5.**
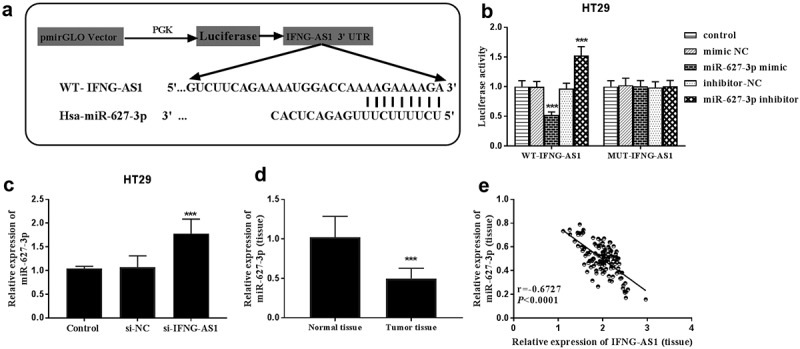
lncRNA IFNG-AS1 acted as a miRNA sponge for miR-627-3p. (a) The predicted potential binding sites of miR-627-3p to lncRNA IFNG-AS1. (b) The luciferase activities of WT-IFNG-AS1 were reversely controlled by miR-627-3p, rather than MUT-IFNG-AS1. ***P < 0.001. (c) Silence of IFNG-AS1 upregulated the expression of miR-627-3p. ***P < 0.001. (d) The expression of miR-627-3p in colon adenocarcinoma tissues. ***P < 0.001. (e) IFNG-AS1 expression was negatively related to miR-627-3p expression. r = −0.6727, *P* < 0.001

DIANA tools LncBase Experimental v.2 predicted the potential target miRNAs. Among the miRNAs, miR-627-3p could suppress the progression of colorectal carcinoma [21,22], and we focused on miR-627-3p. The binding sites between lncRNA IFNG-AS1 and miR-627-3p were shown in Figure 5(a). Subsequently, dual-luciferase reporter assay results indicated that the luciferase activities were decreased by miR-627-3p overexpression, but they were increased by silence of miR-627-3p in WT-IFNG-AS1 HT-29 cell groups (*P* < 0.001, Figure 5(b)). Whereas, the luciferase activities in the MUT-IFNG-AS1 group had no changes. Moreover, the expression of miR-627-3p was evaluated in HT-29 cells transfected with si-IFNG-AS1 by RT-qPCR assay, and miR-627-3p expression was appraised in silence of IFNG-AS cells (*P* < 0.001, Figure 5(c)). Based on the negative correlation, the expression of miR-627-3p in colon adenocarcinoma tissues was analyzed. A decreased expression of miR-627-3p was observed in tumor tissues rather than normal tissues (*P* < 0.001, Figure 5(d)). Pearson correlation analysis results showed that IFNG-AS1 levels were negatively correlated with miR-627-3p expression levels (Pearson correlation coefficient = −0.6727, P < 0.0001, Figure 5(e)). All these data further confirmed that IFNG-AS1 can sponge to miR-627-3p.

## Discussion

In recent years, a large number of lncRNAs related to colon cancer have been discovered, and some of them can be viewed as molecular markers for the early diagnosis and prognosis of tumors [23]. Most colorectal carcinomas in humans appear to originate from adenoma through the process of genetic alterations events [24,25]. In this study, the serum of IFNG-AS1 expression level was firstly detected in colon adenoma, adenocarcinoma, and healthy control. The increased IFNG-AS1 expression was observed in both colon adenoma and adenocarcinoma patients compared to control samples. Moreover, in adenocarcinoma, expression levels of IFNG-AS1 were higher than controls. IFNG-AS1 was reported to have diagnostic potential in several diseases, such as brucellosis [26]. Through analysis of the ROC curve, IFNG-AS1 might be a potential marker for detection of both colon adenoma and adenocarcinoma patients from healthy individuals, as well as colon adenocarcinoma patients from colon adenoma patients, which may offer a novel approach to colon adenocarcinoma diagnosis. In other diseases, like rheumatoid arthritis, elevated expression of IFNG-AS1 in the peripheral blood was observed and had a value of the potential diagnostic biomarker [27]. In coronary artery disease patients, circulating lncRNA IFNG-AS1 expression was also increased and associated with increased disease risk [28].

Subsequently, the role of IFNG-AS1 in colon adenocarcinoma was investigated. The expression of IFNG-AS1 was firstly tested in the tumor tissues of colon adenocarcinoma patients and it could be seen that IFNG-AS1 expression was also climbed in tumor tissues rather than normal tissues. Through clinical analyses, IFNG-AS1 expression was correlated with big tumor size and high TNM stage. Patients with high TNM stage (III–IV) had a higher IFNG-AS1 expression level than low TNM stage (I–II). These findings replied that IFNG-AS1 might play a promoting role in colon adenocarcinoma. The high expression level of IFNG-AS1 was also reported in breast cancer tissues and had diagnostic value for the identification of breast cancer status [29]. Beyond that, Kaplan-Meier and Cox regression analysis manifested that high IFNG-AS1 was bound up to shorter overall survival time and was an independent prognostic risk factor. These findings indicated that IFNG-AS1 might be a potential prognosis marker in colon adenocarcinoma.

LncRNAs get involved in the pathogenesis and progression of malignant tumors [30,31]. Increased expression of IFNG-AS1 could facilitate HP75 cell proliferation, cell invasion, migration, and suppressed cell apoptosis in pituitary adenomas by regulating epithelial splicing regulatory protein 2 (ESRP2) [14]. A recent study demonstrated that IFNG-AS1 expression was increased in acute leukemia patients who received bone marrow transplantation [32]. In this study, the silence of IFNG-AS1 weakened HT-29 and SW480 cell viability, migration, and invasion of colon adenocarcinoma, suggesting that IFNG-AS1 may be an oncogene in colon adenocarcinoma. After that, miR-627-3p was a candidate target miRNA of IFNG-AS1. miR-627-3p was poorly expressed in colon adenocarcinoma tissues. miR-627-3p was identified with tumor-suppressing roles in several tumor types, such as osteosarcoma [33] and lung cancer [34]. Moreover, miR-627-3p could reversely regulate DnaJ Heat Shock Protein Family (Hsp40) Member C2 (DNAJC2) and foster a tumor-inhibiting role in colorectal cancer [35]. Hereby, IFNG-AS1 expression was reversely correlated with the expression of miR-627-3p. Since miR-627-3p was downregulated in colon adenocarcinoma, it is believed that decreased miR-627-3p was involved in the oncogenic activities mediated by IFNG-AS1 in colon adenocarcinoma.

## Conclusion

All in all, the current study demonstrated that serum and tissue IFNG-AS1 expression is elevated as an oncogene in colon adenocarcinoma. The serum IFNG-AS1 has diagnostic value in differentiating colon adenocarcinoma patients from adenoma patients as well as healthy individuals. The tissue IFNG-AS1 showed prognostic significance in post-operative colon adenocarcinoma patients. The silence of IFNG-AS1 repressed the tumor proliferation, invasion, and migration in colon adenocarcinoma cells in vitro by sponge to miR-627-3p. The present study provided potential novel lncRNA-directed early diagnosis, prognosis, and therapy of colon adenocarcinoma, as well as a possible regulatory mechanism of IFNG-AS1 in colon adenocarcinoma.

## Highlights

(1) The serum and tumor IFNG-AS1 in colon adenocarcinoma was upregulated.

(2) IFNG-AS1 might be a potential diagnosis or prognosis predictor for colon adenocarcinoma patients.

(3) Knockdown of IFNG-AS1 could repress malignant progression of colon adenocarcinoma by regulating miR-627-3p.
